# Where you begin is not necessarily where you end: the mental and physical health trajectories of cancer caregivers over time

**DOI:** 10.21203/rs.3.rs-3513142/v1

**Published:** 2023-11-07

**Authors:** Maureen Wilson-Genderson, Maria D Thomson, Laura A Siminoff

**Affiliations:** Temple University; Virginia Commonwealth University; Temple University

**Keywords:** cancer, cancer caregiving, caregiver mental health, caregiver physical health, oncology, primary caregiver, secondary caregiver

## Abstract

**Purpose:**

Cancer caregiving, a critical component in the cancer-care model, has deleterious effects on the caregiver’s physical and mental health. The degree to which these negative effects are uniformly experienced by caregivers is unclear. The impact of the secondary caregiver’s absence on the primary caregivers’ well-being is understudied.

**Methods:**

Terminal cancer patient-caregiver dyads (n = 223) were recruited from oncology clinics and followed for six months or until patient death. Longitudinal latent growth models were used to characterize the heterogeneity of caregiver physical health and depressive symptoms; characteristics associated with these trajectories are examined.

**Results:**

Caregivers were majority female (74%), white (55%) and patient spouses (60%). Two physical health (moderate, stable; initially good, declining) and two depressive symptom (moderate, stable; high, increasing) trajectories were identified. Declining physical health was more likely among caregivers who were healthiest at baseline, had higher levels of education, lower subjective burden, fewer depressive symptoms, cared for patients with fewer functional limitations and reported fewer caregiving tasks rendered by a secondary caregiver. Those with increasing depressive symptoms were more likely to be white, patient’s wife, have higher subjective caregiver burden, lower physical health, and care for a patient with greater functional limitations.

**Conclusions:**

Decreasing physical health was evident among caregivers who were initially healthier and reported less assistance from secondary caregivers. Increasing depression was seen in white, female spouses with higher subjective burden. Sample heterogeneity revealed hidden groups unexpectedly at risk in the primary cancer caregiver role to which the oncology care team should be alert.

## Background

Many aspects of cancer care have shifted from inpatient to outpatient settings in recent decades. The result is burgeoning numbers of informal caregivers (unpaid family, friends) providing care, ranging from transportation to complex clinical care tasks [1–3]. The magnitude of these responsibilities continues to grow with the chronicity of many cancers. Nonetheless, the experiences of cancer patient caregivers (CG) cannot be uniformly characterized due to the wide variations in patient characteristics [4, 5] and caregivers themselves are a nuanced and heterogeneous group [6]. Although early evidence suggested improvements in cancer caregiver physical and mental health over time [7], other evidence suggests that providing informal cancer care has negative physical and mental health consequences for the caregiver [8–14].

The most commonly reported caregiver physical health complaints include sleep disturbance, fatigue, pain, loss of physical strength and weight [8, 9]. In contrast to physical challenges experienced by CGs, those who assume the role are physically healthier than those who do not. A prospective study of community dwelling older individuals identified good physical health as a predictor for becoming and staying a caregiver [15]. Similarly, individuals who became caregivers scored slightly better on physical health than the general US population; however their health declined over a 6-year period [10]. Others have reported similar findings [12, 13]. Together, these findings suggest that good physical health can influence the caregiver self-selection process, but that caregiving is associated with deteriorating physical health long-term.

Mental health can also be negatively impacted by providing care. Depressive symptoms and depression are common; as many as two in five cancer caregivers have depressive symptoms [16, 17]. Predictors of poorer mental health include sex (female), younger age, caring for a spouse, duration of caregiving and decreased access to caregiving resources (insurance, social support) [16, 18–20]. Caregiver burden has been linked to depressive symptoms cross-sectionally [9, 16] and longitudinally [21]. It is noteworthy that caregivers’ experiences of these symptoms may not be uniform and distinct depressive-symptom trajectories exist during end-of-life care [22].

Many caregivers juggle competing demands including employment, childcare, and their own physical and mental health. Some cancer caregivers have social, personal, and economic resources from which to draw while others possess limited resources [23]. Most studies on the caregiving experience have focused on primary caregivers, seldom on the presence or absence of non-primary caregivers which can be characterized as another type of resource. Lund [5] reported that approximately half of the adult cancer patients in their sample identified the presence of a secondary caregiver while Lee [24] found that approximately 40% of cancer caregivers were ‘solo’ caregivers.

Prior work by our research group has examined longitudinally the physical and mental health of primary caregivers with and without secondary caregivers. This study [11] interviewed 171 hematological cancer patient-caregiver dyads (any stage) every 6 months for 2 years. The majority (65%) reported a secondary caregiver. At baseline, supported primary caregivers had significantly lower (worse) self-reported physical health which remained stable. Unsupported primary caregivers had significantly higher physical health at baseline, but their physical function declined significantly over time. While mental health scores were similar at baseline for supported and unsupported primary caregivers, only supported primary caregivers showed improvement overtime. Subsequent modeling indicated that supported primary caregivers’ mental health buffered their physical health; no such effect was detected for unsupported primary caregivers. These results illustrate that caregivers who do not report the presence of a secondary caregiver are more vulnerable to declining health over time.

Given that the trajectory of caregivers’ physical and mental health has been shown to be heterogenous over time [11, 22] and that those with good physical health are more likely to be CGs, the current study will add to what we know by examining the following aims: 1) Evaluate prospectively the physical and mental health of a sample of cancer caregivers for evidence of heterogeneity; 2) Determine factors associated with caregiver health outcomes (e.g., burden, demographics); 3) Examine whether the presence or absence of a secondary caregiver or the amount of caregiving provided by a secondary caregiver differentiate groups of caregivers identified in Aim 1.

## Methods

This was a multi-site, longitudinal, prospective cohort study that followed 223 caregiver-patient dyads. All patients had terminal diagnoses and were recruited from oncology practices in Pennsylvania and Virginia. Dyads were interviewed biweekly for up to six months or until one month after patient death. Data was collected bi-weekly and in-person from caregivers in their homes using semi-structured, in-depth qualitative interviews, quantitative surveys, monthly structured observation, and weekly diary completion. Patients completed a brief survey. All dyads participated in a baseline (T1) interview, an initial observation and up to 11 subsequent waves of data collection. Dyads participated in an average of five (range 1–11) interviews.

### Participant Inclusion and Exclusion Criteria.

Eligible cases were the primary caregivers, at least 25 years of age, of adult home dwelling patients with metastatic (stage IV disease) solid tumors for which the median overall survival was < 12 months and had failed first line therapy (confirmed by oncologist). Patients were considered home dwelling even if they experienced brief periods of hospitalization. A primary caregiver was defined as an unpaid individual who assumes responsibility for assisting and meeting the patient’s daily needs. CGs were self-identified by patients and participation by both CG and patient was an eligibility criterion. All participants provided written informed consent. The study was approved by the Temple University and Virginia Commonwealth University institutional review boards for human subjects (IRB #22776).

### Participant Recruitment.

Patients were identified through the electronic medical record and case conferences from three oncology clinic sites in Virginia and Pennsylvania. A HIPAA waiver of authorization was obtained for patient eligibility identification purposes. Patient oncologists were contacted to confirm eligibility and request permission to contact the patient. Patients were screened by telephone to confirm eligibility including the presence of a primary CG. Of the 875 patients contacted, approximately 23.9% declined participation, 50.3% were ineligible, and 25.4% provided informed consent (51.3% of eligible consented). The baseline sample was comprised of N = 223 dyads. Patients were ineligible primarily due to lacking a caregiver or dying prior to enrollment.

### Data Collection and Measurement.

Cancer diagnosis and stage were extracted from the patient’s medical record. Patient and CG sociodemographic data were obtained directly from participants. At each time point, quantitative measures were verbally administered by Research Assistants (RAs) to patient and CG who were each interviewed privately. Patient interviews were brief (< 10 minutes) and CG interviews were semi-structured (about 40 minutes). All interviews were audio-recorded and transcribed verbatim. The current analysis is based on all 11 waves of caregiver survey and patient medical data.

## Measures

*Demographics* included patient and caregiver age, sex, race, education, annual household income, marital status and caregiver relationship (spouse, parent, offspring, friend).

*Subjective Caregiver Burden* was measured using the Zarit Burden Inventory (ZBI-12) [25] a short version of the original 22-item ZBI (ZBI-22) [26]. It uses a five-point Likert scale, “0 – never” to “4 – nearly always” and can be administered in 3 minutes. The ZBI-12 has good internal consistency with a Cronbach’s alpha of 0.88 [25]. Scores on the ZBI-12 range from 0–48; higher score indicate higher levels of burden.

*Objective Caregiver Burden* was assessed to capture the type, quantity, scope, and intensity of tasks. These were captured in a bi-weekly diary using a 24-hour recall of the Activities of Daily Living (ADLs) and Independent Activities of Daily Living (IADLs) instruments [27]. The original ADL and IADL items are based on validated items from the Index of ADL [28] and the IADL Scale [29] and the addition of cancer-specific items.

*Primary and Secondary Caregiving Tasks*. Caregivers were asked to report if they or another family member performed any of the following tasks in the past two weeks: shopping, food preparation, bathing, eating meals, cleaning/housekeeping, laundry, travel by car/bus, grooming, finance, dressing, getting around inside the home, getting around outside the home, getting in or out of bed/chair, getting on or off the toilet. Items endorsed as being enacted by another family member were summed to create a secondary caregiver task count.

*Presence of a Secondary Caregiver*. If the primary caregiver reported that another family member performed any of the caregiving tasks listed above, they were categorized as having a secondary caregiver.

*Patient Symptom Burden* was assessed using the Edmonton Symptom Assessment System (ESAS) [30], a well-established 10-item self-administered visual analogue scale designed to measure end of life patients’ symptom burden (Cronbach’s alpha = 0.79) [31]. Scores range between 0–90; higher scores reflect greater impairment.

*Self-rated Health*. Caregiver self-rated health was assessed with a single item. ‘In general, would you say your health is currently” rated from 1 (excellent) to 5 (poor) reverse scored so higher scores represent better health.

*Depressive Symptoms*. Depressive symptoms were assessed with the Center for Epidemiologic Studies Depression Scale (CES-D- 10) [32]. Each item was scored on a four-point scale ranging from none of the time (0) to most of the time (3). Positively worded indicators were reverse scored so higher scores indicate higher levels of depressive symptoms.

## Statistical Methods

Descriptive information on demographic characteristics is presented for patients and caregivers. Patient type of cancer, caregiver household income, patient symptom burden, count of secondary CG tasks (past two weeks), presence/absence of a secondary caregiver are similarly presented. We examined means and standard deviations for physical health and depressive symptoms scores at each wave and created empirical growth plots for the respective scores. We used latent class growth models (LCGM) to simplify the heterogeneous set of physical health and depressive symptoms trajectories into more homogeneous clusters. LCGM is used to identify distinct subgroups following a similar pattern of change over time [33].

We built LCGM with one to three classes; we selected the solution with the optimal number of classes, using the Bayesian information criteria (BIC) [34, 35]. We risk-adjusted the LCGM for patient death. Trajectory plots present each LCGM class to permit examination of the average pattern of change. Classes are presented with 95% confidence intervals (CI); if the CI do not overlap there is increased confidence that the groups are meaningfully distinct.

We tested the linear models for the slope of the physical health and depressive symptoms to assess if there was significant change over time and the pattern of that change for each of the LCGM groups; results are reported with the parameter estimate (b) and p-value assessing the significance of the parameter estimate at p-value < .05. The LCGM groups (physical health and depressive symptoms separately) were compared on CG age, patient age, CG education, CG sex, relationship to patient, whether CG was caring for anyone else, count of secondary CG tasks, presence/absence of a secondary caregiver, IADL, ADL, CG race, patient functional limitations (ability), subjective burden and caregiver physical health (for depressive symptom groups) and caregiver depressive symptoms (for physical health groups). Analyses were implemented using SAS 9.4 (SAS Institute Inc. 2015. SAS. ^®^. 9.4) and proc traj.

## Results

### Sample Characteristics.

Our baseline sample consisted of n = 223 CGs who were primarily female (73.9%), white (54.9%), with mean age of 56 years (18–87) (Table 1). A small majority of patients were male (50.9%), white (50.9%), with average age of 62 years (25–94). A plurality of patients were married (46.8%) followed by divorced (26.1%), never married (14%) and widowed (13.1%). The most common relationship to the patient was spouse (45.3%) followed by offspring (22.8%). The most common diagnosis was lung cancer (25.7%). Ninety-seven (43.5%) patients died during the study. The average number of secondary caregiver tasks was 2.15 (sd = 3.08; range 0–13); 53.8% of primary caregivers reported the presence of a secondary caregiver.

## LCGM Results for Self-Rated Physical Health

**Figure 1:**
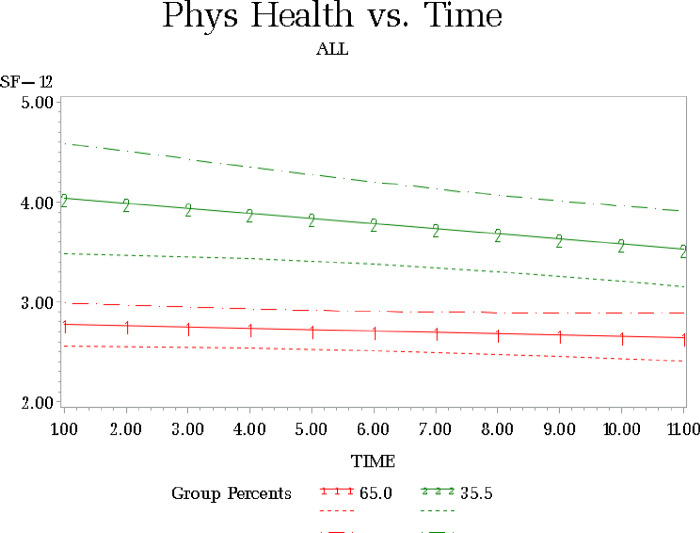
LCGM Cancer Caregiver Physical Health Over Time Group 1 (Stable Physical Health) N = 109; Group 2 (Declining Physical Health) N = 79

The BIC values for physical health solutions with 1–3 classes adjusting for patient death were: BIC= (1) −2140.84; (2) −1938.65; (3) −1934.56. We selected the two-class solution (Fig. 1) as the BIC was comparable to the three-group solution and the latter solution had a group containing less than 5% of the sample [34]. Group 1 (Table 2) contained 65.0% of the sample and had mid-range physical health scores (intercept of 2.8). The linear parameter was not significant (b = − .013, p > .25). We refer to this as the stable physical health group. Group 2 contained 35% of the participants with high baseline physical health scores (intercept of 4.14). This group evidenced a significant linear decrease (b = − .06, p < .005) in physical health status over time. We refer to this as the declining physical health group. Patient death during the course of the study was not significantly associated with membership in either physical health group.

Group 1 (Stable Physical Health) N = 109; Group 2 (Declining Physical Health) N = 79

The physical health LCGM groups were not significantly different from each other on CG age, patient age, CG sex, relationship to patient, whether CG was caring for anyone else, presence of a secondary caregiver, CG employment, IADL, ADL, and race.

Physical health groups differed on CG education, subjective burden, secondary CG tasks, patient symptom burden and depressive symptom scores. Caregivers in the declining physical health group had greater attained education (F (1) = 5.5, p < .02; 3.8 vs 3.2), lower subjective burden (F (1) = 13.8, p < .0003; 9.3 vs 13.0), cared for patients with lower levels of symptom burden (F (1) = 2.94, p < .05; 30.0 vs. 34.0) and lower levels of depressive symptoms (F (1) = 22.8, p < .0001; 6.1 vs 10.1) compared to those in the stable physical health group. Caregivers in the declining physical health group also reported fewer caregiving tasks rendered by a secondary caregiver (mean tasks = 1.6) compared to the caregivers in the stable physical health group (mean tasks = 2.6) (F (1) = 2.79, p < .05). Because this contrast of means failed the assessment of equality of variances between compared groups, we also compared physical health groups for receipt of help from a secondary caregiver on multiple caregiving tasks. The declining physical health group was significantly less likely to report multiple caregiving tasks performed by a secondary caregiver (χ^2^ (1) = 3.2, p < .05; 36.0 v. 50%) compared to the group with stable physical health.

## LCGM Results for Depressive Symptoms

**Figure 2:**
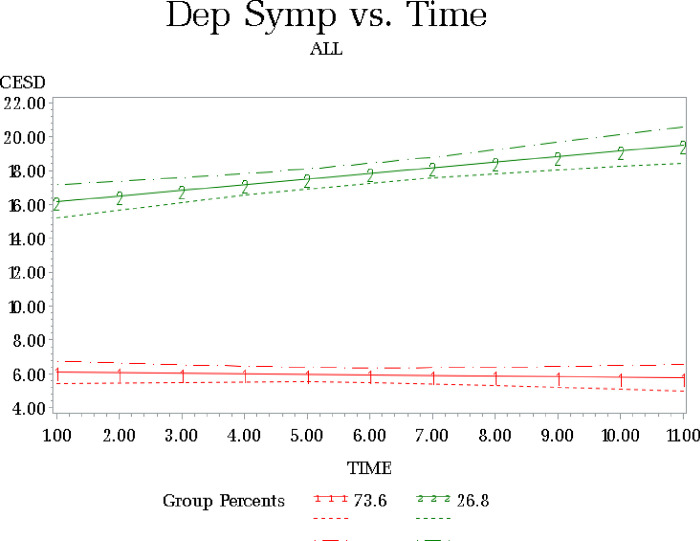
LCGM Cancer Caregiver CESD Over Time Group 1 (Low Depressive Symptoms) N = 141; Group 2 (Increasing Depressive Symptoms) N = 44

The BIC values for depressive symptom solutions with 1–3 classes adjusting for patient death were: (1) BIC=−4965.41; (2) −4357.22 (3); −4365.41. As the three-class solution yielded a singularity convergence error we selected the two-class solution (Fig. 2); which had the lowest BIC and achieved convergence. Group 1 (Table 2) contained 73.6% of the sample and had low depressive symptom scores (intercept of 6.00); we refer to this as the stable mental health group. The linear parameter was not significant (b = − .035, p > .44). Group 2 contained 26.8% of the participants with an intercept of 15.85 and a significant linear increase (b = .33, p < .0001). We refer to this as the declining mental health group. Patient death during the study period was significantly associated with membership in the declining mental health group; more patients of the caregivers in this group died during this study as compared to the group with stable mental health scores (38% v 30%; p < .008).

Group 1 (Low Depressive Symptoms) N = 141; Group 2 (Increasing Depressive Symptoms) N = 44

Mental health (MH) LCGM groups were not found to be significantly different on CG age, patient age, CG education, CG sex, whether CG was caring for anyone else, presence of a secondary caregiver, number of tasks completed by secondary CGs, number of IADL or ADL’s (data not shown). Differences between the stable versus declining mental health groups were identified by race, relationship to patient, subjective burden, cared for patient’s symptom burden and CG physical health. Of the caregivers in the declining MH group, 75% (n = 33) were white and 25% (n = 11) were Black (F (1) = 6.96, p < .009). Caregivers identified as the patient’s wife were more likely to be in the declining mental health group (χ^2^ (1) = 6.2, p < .02; 47.7% vs 27.7%) compared to other relationship roles. Declining mental health was also associated with significantly higher subjective caregiver burden scores (F (1) = 45.9, p < .0001) compared to the stable MH group (17.1 vs. 9.7). Those with decreasing MH reported lower caregiver physical health ratings (F (1) = 18.1 p < .0001 (2.7 vs. 3.4) and cared for patients with greater symptom burden (F (1) = 8.9, p < .003) (ESAS 39.5 vs 30.0).

## Discussion

We prospectively followed end-of-life cancer caregivers and examined the heterogeneity of their physical and mental health. Two distinct groups emerged revealing stable or declining physical or mental health. Similar to values published elsewhere [36], overall mean of self-rated physical health ranged from 3.0 to 3.2 over time. Caregivers who reported declining physical health had better than average physical health at baseline (4.1); they also reported receiving less assistance from secondary caregivers. Decreasing physical health was more likely among caregivers with higher levels of education, lower subjective burden, lower levels of depressive symptoms and cared for patients with lower levels of symptom burden. Mental health was more likely to decrease over time for caregivers who were white, the patient’s wife, had higher subjective caregiver burden, lower physical health at baseline and cared for patients with greater symptom burden.

In the current study we empirically segmented the end-of-life caregiver sample on physical and mental health over time using latent growth curve modeling. Caregivers who began the study with the best physical health and experienced significant declines were less likely to report care tasks performed by a secondary informal caregiver. This group of CGs was also more likely to be caring for a patient with better functional status. This and a previous study of hematological cancer patients have found unsupported caregivers more likely to report substantially better self-rated physical health at base-line declined over time [11]. We hypothesize that CGs who are healthy and initially caring for patients with better functional status may be perceived by their immediate social network as having less need for additional support. These caregivers may also have less available support or be less likely to request support. However, the toll on these unsupported caregivers’ physical health suggests that their need is significant. A recent qualitative study reported cancer caregivers implicitly expect other family members to offer support without having to directly ask [37].

Similar to other recent studies [38] we found that Black CGs were less likely to report high burden or declines in mental health as compared to their white counterparts. In our current study, white women who were married to the patient reported greater perceived caregiver burden and increasing depressive symptoms over time suggesting that resiliency of CGs may be, at least partly based in expectations and life experiences. Black CGs, individuals with less education and economic advantages may not come into the cancer journey with high expectations or may have greater life experiences in coping with personal tragedy and be better equipped to more effectively leverage informal networks of social and resource support. In an older review of the literature, Stenberg et al., concluded that “There is also a paucity of information on the relationships between demographic characteristics (e.g. ethnicity, gender, family structure, socioeconomic status, and cultural heritage), and problems and burdens”[8]. Our results suggest that this remains true.

A recent Cochrane systematic review [39] suggests that existing psychosocial interventions may have little to no effect on cancer caregiver depression and physical health, concluding that rigorous trials with process evaluations and clearer, more detailed intervention descriptions are required. Existing interventions may have failed to identify CGs most at risk and what type or combination of interventions would be effective [40].

## Study Limitations

This study has limitations. The caregiver sample is socioeconomically heterogeneous but skewed towards advantage given that nearly 23% earn over $100,000. The patient sample is heterogenous with regards to type of cancer diagnosis; it is not known if specific types of cancer bring greater amounts of physical and emotional challenges to primary caregivers. The measures of caregiver physical and mental health are self-report, albeit with measures well validated in the literature. It is not known if the results would be different if either were assessed clinically over time. A larger sample might have detected additional groups of physical and mental health trajectories over time in a larger sample.

## Conclusions

Our work has shown that there is a significant minority of caregivers who are vulnerable to either physical and/or mental health decrements associated with the provision of informal cancer care. Long identified as ‘hidden’ patients with concomitant calls for attention to caregiver health and unmet needs [39, 41, 42] our results highlight the urgency to assist cancer caregivers as declines were found over a short end-of-life period. Caregivers who become sick cannot continue to provide care and may develop significant and costly healthcare needs themselves with implications for their future employment, financial security, and quality of life. This study and others suggest that identifying subsets of CG characteristics and their specific vulnerabilities is required to develop effective, targeted interventions.

## Figures and Tables

**Table 1:** Patient and Caregiver Sociodemographics

	PT		CG	
	N	%	N	%
**Female**	111	49.12	167	73.89
**Hispanic**	7	3.1	4	1.77
**Race**				
White or Caucasian	115	50.88	124	54.87
Black or African American	91	40.27	90	39.82
Other	7	3.09	2	0.88
Multi-racial	6	2.65	7	3.1
American Indian or Alaska Native	4	1.77	.	.
Asian	3	1.33	3	1.33
**Age (M, SD)**	61.62	11.66	55.74	13.87
**Cancer**				
Lung	58	25.66		
Pancreas, liver and biliary	33	14.6		
Colorectal	29	12.83		
Bladder, kidney (renal cell)	22	9.73		
Breast	19	8.41		
Ovarian, endometrial, uterine	15	6.64		
Head, neck, throat	13	5.75		
Gastric, esophageal	11	4.87		
Prostate	11	4.87		
Other	15	6.63		
**Education**				
Less than high school degree	52	23.01	22	9.73
High school diploma or GED	57	25.22	52	23.01
Some college/Associates degree	75	33.18	89	39.38
Bachelor's degree	20	8.85	33	14.6
Postgraduate work/degree	20	8.84	29	12.83
NR/NA	2	0.88	1	0.44
**Relationship to Patient**				
Spouse			101	45.3
Offspring			51	22.8
Sibling			28	12.6
Parent			14	6.25
Other			29	13.0
**Household Income**				
Less than $50k			99	44.4
$50k-$100k			73	32.8
GT $100k-$150			22	9.9
GT $150			29	12.9

**Table 2:** LCGM Depressive Symptoms and Self-Rated Physical Health Two-Class Solutions with Change Parameters

		Group 1	Group 2
**CESD**		*beta (se)*	*beta (se)*
	Intercept	6.00 (.30)	15.8 (.57)
	Linear	− .035 (.05)	0.33 (.08)****
	Died	[referent]	0.40 (0.15)**
**Physical Health**		**Group 1**	**Group 2**
	Intercept	2.8 (0.11)	4.14 (.22)
	Linear	−.013 (.01)	− .06 (.02)**
	Died	[referent]	−0.17 (.22)
